# Re-invigorating the photo album: augmenting printed photobooks with digital media

**DOI:** 10.1007/s00779-022-01699-5

**Published:** 2022-11-24

**Authors:** Emily Corrigan-Kavanagh, David M. Frohlich, Caroline Scarles

**Affiliations:** University of Surrey, Guildford, GU2 7XH UK

**Keywords:** Human-centred computing, Human–computer interaction (HCI), Interaction paradigms, Mixed/augmented reality, Storytelling, Photobooks, Travel, Tourism, Augmented paper, Memory, Identity, Self-representation, Socialisation

## Abstract

The photo album emerged in the late 1800s as place to collect portrait photos of visitors to a home, and was later appropriated by Kodak as a visual chronology of family history. With digital photography, the album has largely been replaced by online repositories of images shared on social media, and the selective printing of photobooks. In this paper, we present a ‘next-generation paper’ authoring system for annotating photobooks with multimedia content viewed on a nearby smartphone. We also report the results of a trial of this system, by nine travellers who used it to make augmented photobooks following a trip. These findings show that the augmented physical-and-digital photobook can heighten awareness of the multisensory aspects of travel, enrich memories, and enhance social interaction around photos. The social and technical implications for the future of the photo album are discussed.

## Introduction

Information and communication technologies (ICTs) have revolutionised how we represent ourselves personally and to others. They provide a plethora of multimedia creation opportunities through the prolific spread and use of smartphones [1] as well as image, audio, and video capture facilities, with internet connectivity to social media websites (i.e. Facebook, Twitter, Instagram). They also afford sharing of individual or combined image, audio, and video recordings with textual messages to a range of audiences, inviting audience response [2]. Digital repositories of such recordings build up on personal devices and cloud services, making it somewhat difficult to find individual items and organise them [3]. Against this backdrop, printed photographs in the form of photobooks are still valued for imposing order and providing a physical memory artefact of lasting value. They complement the volatile nature of digital media which can grow, change, or vanish over time [4].

In fact, professional looking photobooks are now easier than ever to assemble, create, design, and obtain [5] through specialised digital authoring tools [6–8]. These tools allow ways of creating conventional photo albums more quickly and professionally than ever before, directly from a smartphone app in some cases. In contemporary life, photobooks appear to mark out some events and experiences as particularly special; elevating their presence from the wealth of digital photographs circulating more routinely on personal devices and social media sites. This echoes the function of traditional analogue photo albums which were the public face of the wider printed photo collection, carefully crafted to portray happy and successful aspects of family life [9].

In parallel, the digital revolution has also led to digital storytelling, in which individuals assemble multimedia narratives of their experiences for others [10, 11]. This goes beyond the sharing of individual media items or even their combination into multimedia collections. The digital story is a multimedia *narrative* akin to a personal film with a beginning, middle, and end, and constitutes a new and powerful form of storytelling beyond the visual narrative of a photo album [12]. The communication of travel experiences is a good example of this where tourists portray travel activities and encounters, using audiophotos, crafted videos, or Instagram stories [13–15].

Augmented reality (AR) can bring together practices of printed and digital storytelling by for example overlaying supplementary multimedia such as text, audio, and video onto tourist sites or museum exhibits [16]. Media can be triggered from photographs, QR codes, geolocation, or RFID tags on a smartphone [17]. In our work on the Next Generation Paper (NGP) project, we have been applying this approach to paper by developing a new platform for augmented books, or ‘a-books’ for short, based on the EPUB 3 format for interactive e-books. Documents can essentially be authored as e-books with additional hidden multimedia, activated by interactive buttons on each page, and then printed to be read with the same multimedia displaying on a nearby smartphone or tablet. We have been applying this technology in the domain of travel and tourism, initially through the design and evaluation of a professional travel guide to Cornwall and the Scilly Isles [18]. In this paper, we describe its application to augmented photobooks (‘a-photobooks’) made by travellers themselves. Specifically, we explore how a new form of *printed digital storytelling* might emerge from this combination of physical and digital, giving new life and meaning to the traditional photo album. We begin by reviewing the evolution of the photo album into conventional and enhanced photobooks. We go on to describe the NGP Authoring and Player apps before reporting a trial of these to create an augmented photobook following a travel experience.

## A brief history of the photo album

The digital camera revolution started around the same time as the internet revolution in the 1990s, and well ahead of camera phone and social media developments in the 2000s. This means that anyone born before about 1980 will remember the age of analogue photography and the photo album. In that period, all photographs were physical prints which cost money and took up space in the family home. Cameras were also expensive and typically shared within a family, leading to photo albums of family rather than personal photography. This is worth re-iterating for younger readers and to underscore the key role of the traditional photo album in curating and sharing family memories. Loose printed photos developed from 24 or 36 exposure films were often double or triple printed to give or mail to others, stick or frame around the home, and slip into photo albums for sharing face-to-face [19]. The album was in effect the primary social media site for analogue photo sharing, reaching only as many people as it could be physically shown or handed to. This continued an even older tradition of carte de visite albums, containing photos of visitors to the home and famous people admired by the household. Carte de visites themselves were a kind of visiting card ‘selfie’ of the 1800s, but taken by portrait photographers to give to your friends [20].

With the advent of digital photography, the volume of photos taken went up but the number that were printed went down. Consumers made a double saving on film and development costs, as long as they viewed digital photos on screen. This could be done on the back of the camera but also on the home PC which became a major site for early photo sharing before the camera phone arrived [21, 22]. The camera phone made the taking, viewing, and communication of photos more convenient in one fell swoop, paving the way for social media systems to handle the huge volume of ‘mobile’ photos that began to circulate [23]. Printing stopped being comprehensive of all photos taken, and became more selective and professional. This opened the door to professional photobook services, alongside a diversity of other photo printing options such as framed photos, posters, tee-shirts, mugs, and mouse mats. The authoring of photobooks was made very easy through dedicated photo websites such as PhotoBox and Cewe, and more recently incorporated into major photo archiving services such as Google Photos or Apple iPhotos. Photobookscan now be created and ordered direct from a smartphone, using dedicated layout apps such as Snapfish, Shutterfly, and Chatbooks. The global photobook market is currently very healthy, at $3.376 billion in 2021, and expected to rise about 3% in the coming years [24]. However, we can find no recent published research on consumer photobook practices, in contrast to the many articles on social media use. There seems to be an assumption in the literature that the photo album has been superseded by online photo sharing, whereas in fact it has found a new photobook form and subsidiary role alongside it.

Innovation in both photo album and photobook design is evident in the research literature and associated products. These include attempts to automate aspects of photobook creation from a collection of digital photos, and to augment them with digital information. For example, Rabbath et al. [4] try to identify story events within a person’s social media history to auto-generate a photobook containing their photos and those of others involved in the event. In another approach, Boll et al. [25] use photo metadata and related online information to add text to photobooks. Henze and Boll [26] also show that readers can query this metadata from a photobook, using the augmented reality (AR) approach of taking a photo of the printed photo. Gaggi and Ghidoni [27] in their *smilingPhotos* system show how an augmented photobook can be created to yield a multimedia presentation as well as a photobook, while Fageth [28] creates a photobook from the frames of video clips and uses printed QR codes to index the source video on a smartphone. The Livescribe pen/camera and Anoto patterned paper is used in two augmented photo album systems, to play associated audio and video clips on a nearby phone [29, 30]. Commercial ‘talking photo albums’ support audio recording and playback through the album itself (https://www.talkingproducts.com), while Shutterfly have experimented with photobooks with up to 30 s of spoken narration per page, indexed by printed QR codes (https://www.shutterfly.com).

All these innovations show various ways in which printed photobooks might be more closely connected to other online media during authoring or reading. However, they use a variety of different technologies and formats, and often fail to show what content or benefits are chosen and experienced as a result. In the rest of this paper, we describe a new authoring and playback system for augmented photobooks using a standard e-book format, and findings of a user study with nine UK-based travellers. Through the user study, we try to understand the perceived benefits of digital augmentation of photobooks and how users would like to use these in the future.

## Next-generation paper platform

On the NGP project, we have been exploring both AR and IoT (Internet of Things) technologies for augmenting paper within the same framework and platform. We refer to these as second-generation (2G) and third-generation (3G) paper respectively, because AR technology is cheaper and more mature than IoT technology at present. In the rest of this article, we concentrate on an AR implementation of augmented photobooks which uses image or speech recognition of ordinary paper to trigger printed hotlinks on a nearby phone. More information can be found on alternative 3G paper technologies from our other publications [31].

**Fig. 1:**
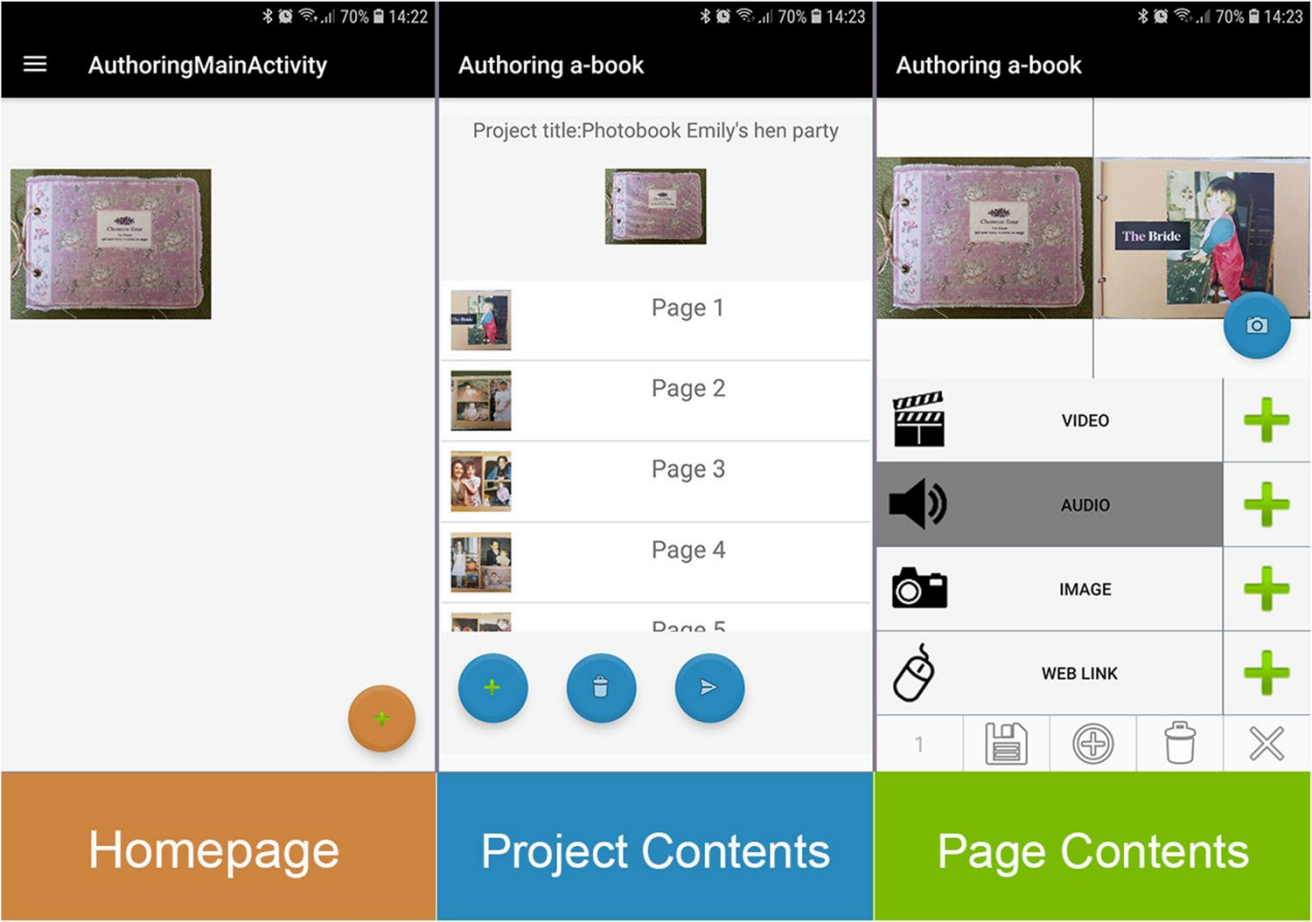
The NGP Authoring app homepage, project contents, and page contents window as it appears on a smartphone device screen, read from the left to right-hand side (authors’ own work)

We have also been applying this approach to both professional and self-made books in the travel domain. Professional travel guide a-books can be created using industry standard book layout tools such as Adobe InDesign. These allow interactive buttons/hotlinks and associated digital content to be embedded in (interactive EPUB 3) versions of e-books for any print book. Our NGP Player app can read these files to playback digital content from a printed book [32]. However, we do not expect travellers themselves to author their own augmented books using professional-level tools like InDesign. Instead, we have created an NGP Authoring app for them to use to annotate an existing printed book, ‘paper-first’. This could apply to any book they happen to already have on their bookshelf, or to a photobook they created in the normal way through photobook creation tools online. Once authors receive their photobook from a print service provider such as Photobox, they can use our authoring app to annotate it with additional digital content from their smartphone. After taking a picture of each page they want to annotate, they press a + button to add an image slideshow, audio recording, video recording, or web URL. Multiple items of each kind can be added to any page. The user interface for these actions is shown in Fig. 1. This differs from previous approaches by (a) using standard sensor and client technology such as a smartphone, (b) supporting multiple layers and instances of links on any page, and (c) supporting the annotation of legacy books or new ones without special printed marks like QR codes.

**Fig. 2:**
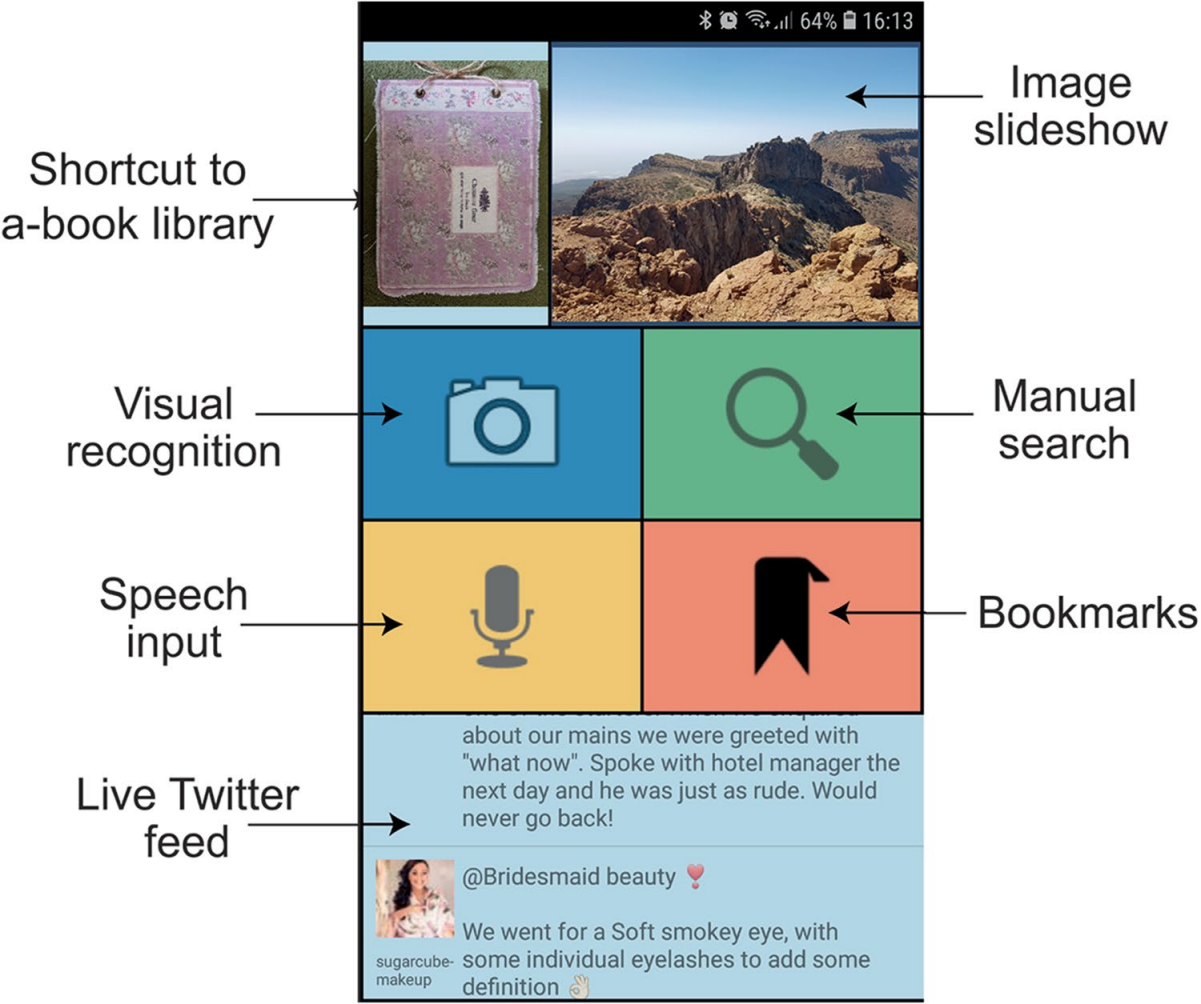
NGP Player app homepage with annotated features as it appears on a smartphone device screen (authors’ own work)

From a homepage showing thumbnail cover images for each a-book ‘project’ being created, users enter a project index page where they can add images of each page of the book. From there they can go to each page and add one or more instances of each of the four link types shown. Image, audio, or video content can be recorded within the app or imported from the smartphone’s respective media galleries. Weblinks can be searched for and selected through a browser. Once all annotations have been added, the user saves the resulting project as an a-book file which can then be imported into the NGP Player app shown in Fig. 2. This is similar to the e-book paradigm where a library of e-books is imported into an e-book reader for reading.

Once the a-book file is imported into the Player app, this file can be opened in the Player app to automatically recognise physical photobook pages and play any of the four link types previously linked to them through the Authoring app. The Player app can recognise pages from the related photobook by the user taking a picture of the page, by manual input of the page number, or by speaking the page number. Associated content is then presented for each page on the smartphone itself as a list of media to be selected and played on screen. Users can also bookmark, and label pages selected that will then show up under the ‘Bookmarks’ button, input-related keywords to set a live Twitter feed, and view and manually swipe through all digital images linked to the photobook using the image slideshow.

As smartphones are becoming a ubiquitous global technology, their use in the creation and playback of a-photobooks through use of bespoke smartphone apps is an obvious strategy with minimal new learning for users. The a-photobook apps are also purpose-built to allow travellers to augment and playback content from their photobooks with little support. This counters common issues of AR uptake where a systematic review of AR in tourism by Yung and Khoo-Lattimore [33] showed this is usually impeded by equipment costs and usability issues, and AR platforms being employed outside their original intended use. Subsequent sections will now present the methods used and emerging findings from creating and evaluating a-photobooks with nine UK-based travellers.

## Methods

### Approach

The study was carried out in the second half of 2019 prior to the Covid-19 pandemic, and involved 1:1 face-to-face and remote interactions with participants. We used a design research approach in which working prototypes were created and given out speculatively for unconstrained use in a small-scale field trial. Details of participants and procedure are given below. Of the nine participants that fully took part in the study, the NGP Authoring and Player apps were installed on their Android smartphones and demonstrated to them before they travelled on a holiday trip. On return they use a Photobox voucher to create a physical photobook of the trip, and attended a co-design workshop with the first author to annotate this with digital media using the Authoring app. In the same workshop, they use the Player app to playback content for reflection and discussion with us. They returned 2 weeks later to discuss social use and sharing of the a-photobook with family and friends. In this way, the Authoring and Playback apps, together with the created a-photobooks, acted as research probes into the values and practices of prospective users to anticipate how they might use and modify the technology in the future [34].

### Participants

12 participants were originally recruited using criterion sampling through university networks, with the following criteria: adult (over 18 years); UK resident; access to Android phone; taking a holiday with family and/or friends in the next 4 months. Three participants dropped out when their holiday plans changed (i.e., taking place beyond the study timescale) or were cancelled, leaving nine who completed the study. This study received full ethically approval by the university’s ethics committee.

### Procedure

We felt it was important to discuss the augmented book technology and affordances with participants before they travelled on holiday. They needed to know that the technology supported the annotation of image slideshows, audio recordings, video recordings, and weblinks from individual pages of the printed photobook and that they could collect these additional materials on their smartphone while travelling, rather than attempting to source them on return. This was done through a quick 15-min one-to-one briefing meeting with each participant where they were also asked about their previous experience of photobooks (i.e., “Have you created a photobook before or do you currently use them? “If yes please tell me about it?”) to better understand their previous experiences and preconceptions about photobooks that might affect the results. They were then emailed downlinks with instructions on how to install both the Authoring App and Player app in their own time but could contact the researcher for further support if they had difficulties with this. Participants were not directly shown how to use apps at this stage as the researcher did not want this to influence or limit the types of multimedia content they captured to help assess how the technology complemented or challenged current multimedia recording practices.

When they returned from holiday, each participant used a Photobox voucher to generate a free photobook of their trip using their online site. Once completed, they took this to the co-design workshop to augmented it with multimedia and web content using the Authoring app installed on their smartphone, under the researcher’s guidance. Before using the Authoring app, participants were first asked to show and explain the meaning behind images in their photobook so that the researcher understood the significance of images they had captured and why they had decided to include them in final photobook. Participants were also asked to explain the multimedia content that they had recorded, such as what was its purpose (i.e., for reminiscing, explaining, planning, general interest?).

Subsequently, they used the Authoring app to augment their photobooks with initial guidance from the researcher. Once they had completed this, using the Player app installed on their device, participants played associated digital content while reading their photobooks. Participants then answered questions about their initial reactions to the technology for the remainder of the workshop. Sample interview questions included:

- What are your initial impressions of the apps? Do you find them user friendly, robust, engaging, or boring?
- Now that you have composed your augmented photobook, how might you and your family and/or partner use it in the future?
- Do you think the augmented photobook will influence how you and your family and/or partner will *record* (i.e., take photos, capture video, audio, collect mementos) your holidays in the future?

Two weeks later, each participant attended a follow-up semi-structured interview to discuss their overall impressions of the a-photobook, such as how they used it and shared it with others, and what they imagined using it for in the future. Example interview questions included:

- How was the augmented photobook used, if at all? (e.g., by who, for what purpose and what multimedia was played?)
- Were there any surprises in how you and your family and/or partner interacted with the augmented photobook? (e.g., new ways of viewing/seeing/imagining places?)

### Analysis

All study sessions were captured using audio-visual footage and based at the university of the research team or over videocall dependant on preference. Video captured the visual context of participants’ interacting with photobooks and/or answers to interview questions, providing embodied context for deciphering underlying emotions to verbal statements. Corbin and Strauss’s [35] grounded theory, using opening and axial coding, and reflective memoing, enabled the conceptualisation of findings around three specific themes (see below). Additional content analysis of the augmented photobooks themselves was conducted to understand the composition of printed and digital content. We begin the results section with a brief characterisation of this content, before stepping through each of the emergent themes from the interviews about it.

## Results

### User experience of authoring app and player app

All nine participants reported the Authoring app to be very user friendly. This was supported by the minimal guidance participants required when augmenting their photobooks during the co-design workshops; all participants only required one example of how to add one type of multimedia content to their photobook before they could complete the full augmentation of their photobook without further assistance. Four participants also remarked that their interaction with the Authoring app encouraged them to think differently and more creatively in how they represented holiday stories using the additional multimedia content such as how audio and video clips could be used to bring their printed photos to life. However, some participants found adding audio and video content confusing as they needed to know in advance and search for the right folder that this was stored in on their smartphone in order to add it to the specific page they were augmenting.

In terms of desired additional features, all participants agreed that the Authoring app should automatically name/add consecutive numbers to pages as they were created instead of requiring the user to do this each time a new page was added. This led some participants to forget the number of the page they were previously on and misnumber the next one when creating their augmented photobooks. Most participants also wished for a group version of the Authoring app to create one collective augmented photobook between multiple individuals where each person could use the app on their smartphone to add content and make changes to one augmented photobook simultaneously.

Most participants (six of nine) reported that the Player app was user friendly. Specifically, more than half remarked that the image recognition feature was useful for quickly retrieving linked multimedia from printed photobook pages, while four identified the bookmark feature as particularly helpful for rapidly searching for and finding multimedia linked content of particular interest. However, none of the participants used the Twitter feed feature and most (eight of nine) did not seem to understand the point of it with four suggesting that it should be removed entirely from the app. Other suggested improvements included adding the ability to input descriptions of pages alongside page numbers when searching for content through the Player app. More than half of participants also wanted the steps in accessing the multimedia content through the app reduced, such as by playing content for each page automatically once it has been selected rather than selecting the link types individually from a displayed list.

### Augmented photobook content

Participants used a range of digital content such as video, weblinks, audio, digital images, and digital image slideshows to augment and add deeper meaning to their personal photobooks as well as extend the social interaction opportunities the physical photobooks afforded. Final a-photobooks ranged in size from 23 to 36 pages, containing between 25 and 161 photos. A total of 41 video clips, 41 weblinks, 20 audio clips, 19 digital images, and seven digital image slideshows (i.e. video of different digital images playing sequentially) were linked to the nine personal printed photobooks created by each of the nine participants. All digital media link types were linked to one separate photobook page that could contain either one image or multiple images (between two and seven). In instances where there was more than one printed photograph per photobook page, all images contained content on the same theme, such as family and/or friends having a meal or playing games, or a place of interest seen from different angles.

Participants mainly preferred to include one link type per photobook page but occasionally would add two different link types to the same page. For example, one participant (R1) added a digital image and a video to the same photobook page to show more contextual information about an art installation, such how it looked, moved, and sounded. Table 1 shows a breakdown of the number of photos and their page distribution in each photobook as well as the different types of digital content linked to each photobook, and examples of the content each contained.

**Table 1:** Participant’s photobook composition and number of digital media types with examples of content used to augment printed photobook pages to create a-photobooks (author’s own work)

Participants (photobook composition)	Link type breakdown			
R1 (25 printed photos over 23 pages, one image per page)	5 video clips (e.g. see view, art installation)	2 weblinks (e.g. accommodation, interesting food)	4 digital images (food from a food festival, art installation)	
R2 (45 printed photos over 26 pages, one image per page on 14 pages, multiple images per page ranging from two to four on 12 pages)	12 weblinks (e.g. historical information on plates of interest) with two weblinks linked to online videos (e.g. sea waves)		1 audio clip of human speech (e.g. relative speaking)	
R3 (54 printed photos over 26 pages, one image per page on 14 pages, multiple images per page ranging from two to seven on 12 pages)	1 video clip (e.g. place of interest)	3 weblinks (e.g. accommodation, restaurant)	4 audio clips, ranging from music to sound effects (e.g. favourite music tracks, sound of someone eating)	7 digital image slideshows (e.g. maps of journey)
R4 (27 printed photos over 25 pages, one image per page)	8 weblinks (e.g. Google map locations) with two weblinks linked to online videos (e.g. running water)		1 audio clip of human speech (participant speaking)	
R6 (161 printed photos over 36 pages, one image per page on four pages, multiple images per page ranging from two to nine on 32 pages)	6 video clips (e.g. pets fighting, seagulls eating bread)	9 weblinks (e.g. accommodation, places of interest visited, restaurants)		6 digital images (participant’s partner, surrounding landscape)
R7 (66 printed photos over 32 pages, one image per page on 14 pages, multiple images per page ranging from two to four on 18 pages)	9 video clips (e.g. participant driving through snow, participant’s friends rolling in snow)		2 audio clips of participant’s activity (footsteps walking through snow)	
R10 (92 printed photos over 36 pages, one image per page on 14 pages, multiple images per page ranging from two to six on 22 pages)	12 video clips (e.g. surrounding area, participants jumping into the sea)	6 weblinks (e.g. tourist and transport sites, places of interest visited)	7 audio clips of sounds from surrounding area (e.g. waves from the sea, planes taking off)	9 digital images (e.g. accommodation, restaurant meal, transport)
R11 (40 printed photos over 23 pages, one image per page on 7 pages, two images per page on 17 pages)	5 video clips (e.g. partner wearing ring, relative reciting alphabet)			
R12 (38 printed photos over 26 pages, one image per page on 17 pages, two images per page on 21 pages)	3 video clips (e.g. shopfronts with same audio narration, surrounding seaside)	1 weblink (e.g. accommodation)	5 audio clips (e.g. background music at accommodation, seaside sounds)	

Table 1 shows that different participants used the link types in different combinations when making their a-photobooks. Video clips were the most popular digital media used by participants with seven of the nine participants linking self-recorded video clips to pages in their photobooks. Notably, the remaining two participants who had not created self-recorded video clips used weblinks to also link (online) videos clips to pages in their photobooks. Weblinks were also popular and used by seven out of nine participants. Audio clips were used by six out of nine participants but tended to be longer than video clips with an average length of 1 min 37 s in comparison to 28 s for video. Digital images were the least favoured digital media with four of nine participants utilising this medium. Digital images employed tended to consist of out-takes relating to the same theme as the printed images in the physical photobook. This allowed users not to ‘waste’ poorer images not good enough to make the printed album, but also to ‘hide’ more private images in the digital links under their control (see Sects. 5.3 and 5.4 below). A summary of typical participant use of audio, video, digital images, and weblinks in resulting augmented photobooks can be viewed in Table 2.

**Table 2:** A summary of participants’ common use of audio, video, digital images, and weblinks to augment their printed photobooks

Participant trends	
Use of weblinks	Mainly consisted of websites of visited places linked to photobook pages with representative photos
Use of digital images	Mainly consisted of out-takes of printed photos on photobook pages
Use of audio clips	Mainly added to provide greater auditory ambience to printed photos on photobook pages
Use of video clips	Mainly added to provide greater visual and auditory context to printed photos on photobook pages

**Fig. 3:**
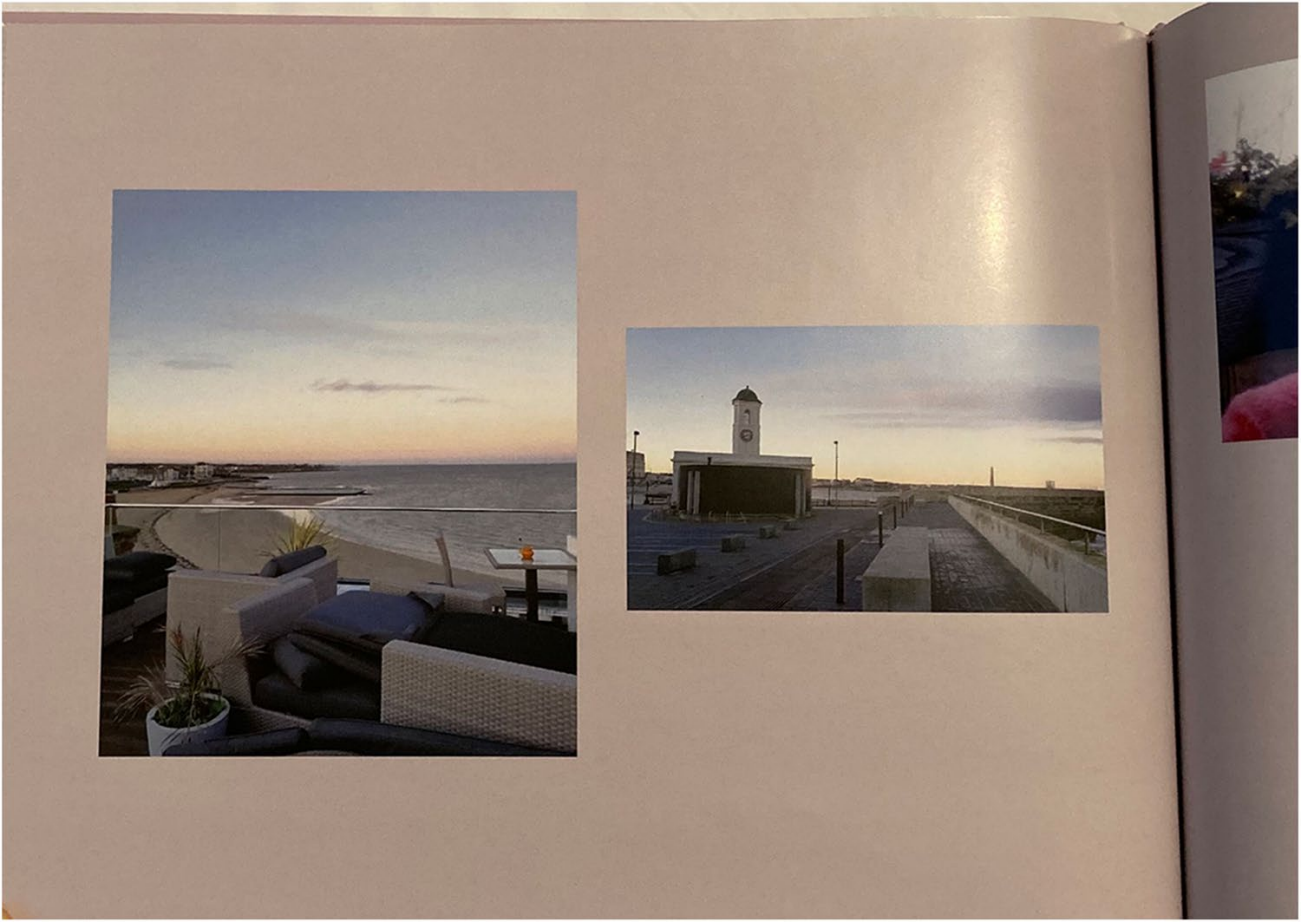
A photobook page with accompanying video of the scene shown on the left

We can also illustrate the typical use of audio, video, and weblinks in relation to the album created by participant R12. This participant travelled to Margate for Christmas 2019 with her teenage daughter. Figure 3 shows Page 20 of the photobook they made from this trip, showing a photo from the roof terrace of their hotel and a clock tower building on the promenade. This is linked to a 30-s video clip panning across the roof terrace with accompanying wind and seagull sounds. It was common for most videos to contain only ambient sound of this type, but some also contain additional voice narration describing the scene or feelings of the narrator. This video clip can be viewed at the following Vimeo page: https://vimeo.com/showcase/9519891/video/709110993. The video clips generally serve to bring the printed photos to life. In fact, they can be seen as a longer version of Apple Live Photos, but split across paper and screen — here 10 times the conventional length of a 3-s Live Photo.

**Fig. 4:**
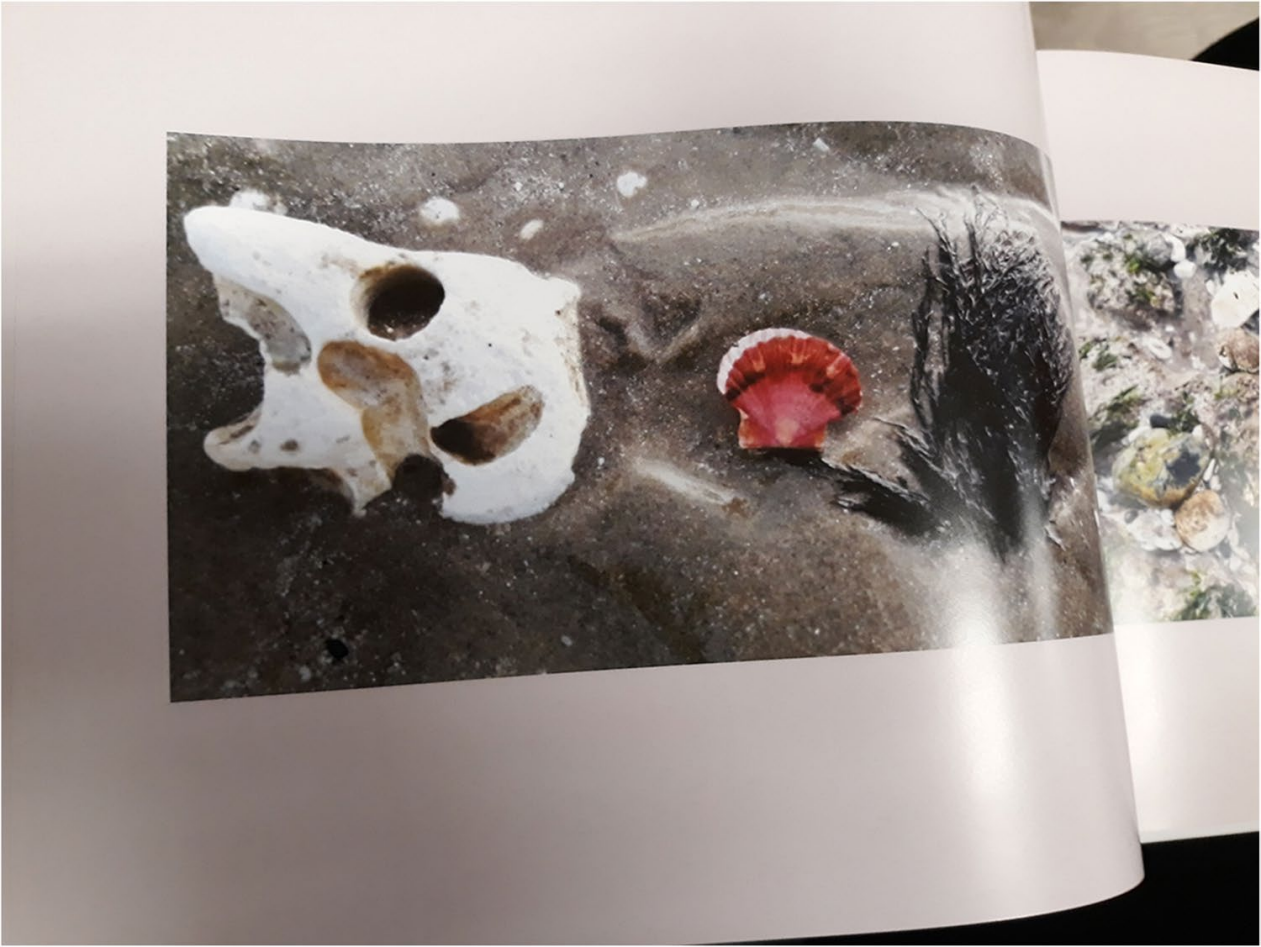
A photobook page with accompanying audio of the beach picture shown

Audio clips were used in a similar way to video to bring the printed photos to life. This is shown in Fig. 4 which shows Page 12 from the same Margate album, ‘illustrated’ with a 52-s sound recording of seagulls and the sea on a walk along the beach on Christmas Eve. It can be played from the following Vimeo page: https://vimeo.com/showcase/9519891/video/709129704. As mentioned above, these audio clips were typically longer than the video clips, and allow an activity or scene to unfold at its own pace in real time. 15 out of 18 audio clips across the corpus were ambient sounds with only 2 used for spoken storytelling over the printed image. The remaining three audio clips were music recordings, as in a recording of ‘Let it snow’ playing in the hotel bar on Christmas Day in this album.

**Fig. 5:**
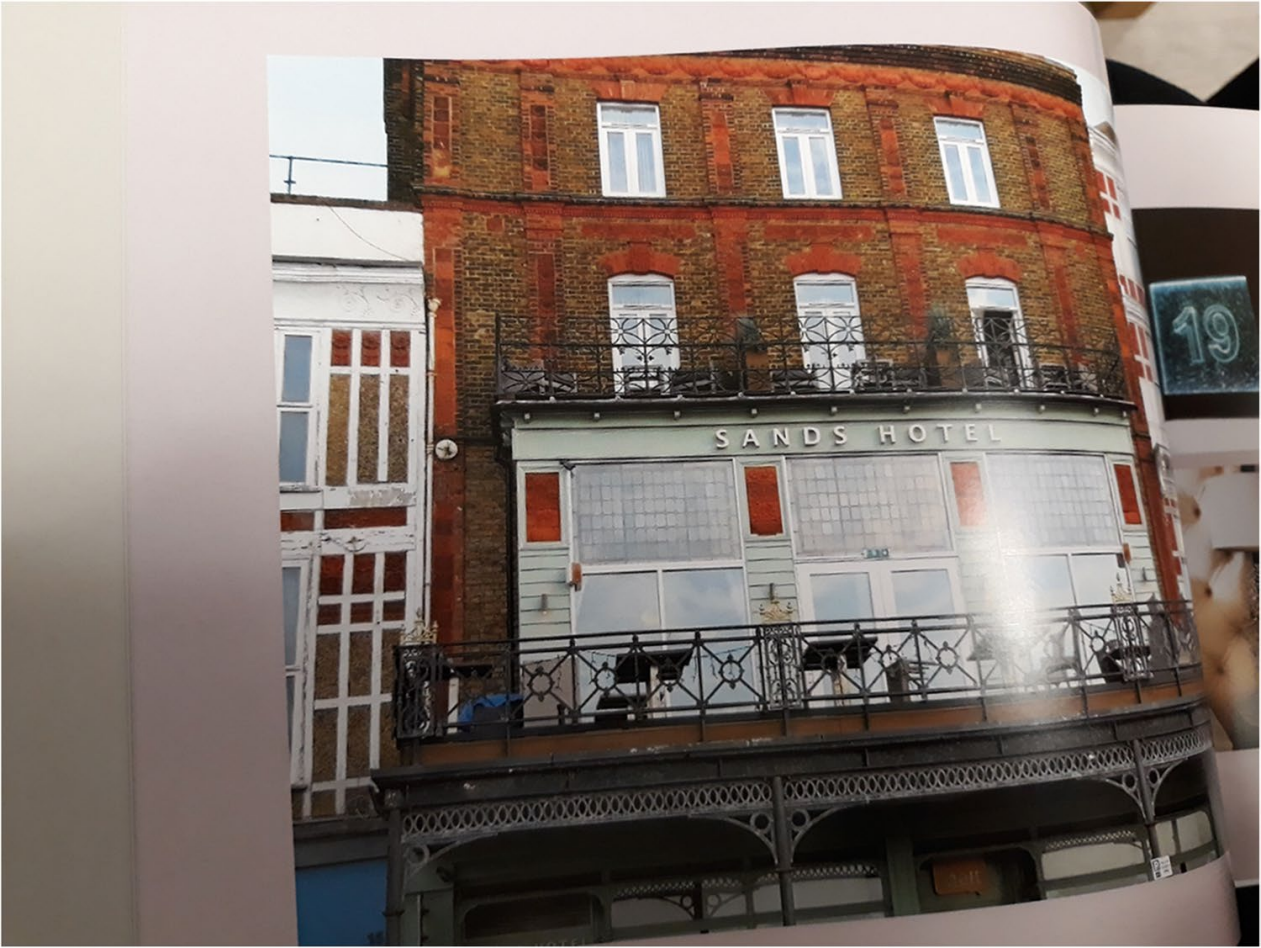
A photo of the holiday hotel linked to the hotel website

Finally, weblinks tended to be used for quite practical purposes to link the printed page with the websites of visited locations. For example, Fig. 5 is Page 4 of the photobook showing a photo of the hotel the participant and her daughter stayed at. This was linked to a website of the hotel: https://www.sandshotelmargate.co.uk. As Table 1 shows, weblinks also included restaurants, heritage sites, museums, galleries, and transport methods. Participants seemed to use these to record details that might be useful to themselves or others later in revisiting the sites.

### Heightened awareness to multisensory experiences

Participants agreed that creating the a-photobook increased immersion in holiday moments as they focused more closely on different sensorial elements to find suitable digital content. Rather than photographers, participants became storytellers, creating narratives of their experiences utilising various mediums, especially video footage as shown in Table 2. Reflecting on the creation of content for the a-photobook while on holiday participant R1 commented, ‘I was a lot more tuned into things I was hearing, the sounds of places, capturing stuff, like filming stuff and capturing stuff with it in mind, thinking oh this would be nice to have…’.

This sensitivity to multisensory experiences was reflected in how resulting a-photobooks were conceived by participants, which could be divided on a ‘personal level’ (i.e. what media could be created to *enhance* the printed photobook), ‘creative level’ (i.e. how the media linked to the photobook could *expand* image-based stories), and ‘social level’ (i.e. how the final photobook content could be *presented* to others). On a personal level, participants reported deeply tuning into their environment to find appropriate multimedia that would enable them to relive their experiences. As put by participant R12, ‘so you may be plugging into the experience in a way that you wouldn’t normally because you know you’re collecting content’. Another participant (R2) reflected on how the media created for the photobook “can capture things [personal experiences] that are lost with just photos… things like a seascape… you cannot capture the sound, but you can combine it through the tool” encouraging participants to consider additional sensory elements (i.e. sound) that they could not ordinarily capture with photos alone.

On a creative level, participants also had to consider how additional digital media link types could be used to expand on the image-based stories represented in the printed photobook. As previously indicated by Table 1, different media types could provide additional contextual information, such as video clips could provide footage of scenes depicted in photobook images. For example, participant R4 reported a growing desire to record audio of relatives speaking in the moment to later link to images of them on photobook pages, justifying this approach with ‘if it’s a still photo then I think the audio content adds more of a story to it, adds more of a personal touch to it’. Similarly, participant R10 spoke about how he used a video clip of him jumping into the sea to show the reality behind a photo of the experience, commenting ‘It was a lot more impressive in my head when I was doing it, [but] when I watched the video back I was like, ooh loser [laughing]’.

On a social level, participants reported tuning into their experiences to identify potential curated media that would communicate the sensory nature of their experiences to others as well as themselves. For example, participant R1 remarked, ‘Like normally you don't want to bore people to death about the details of your holiday… I was really kind of curating what I was taking and thinking about it, rather than being snap happy’. Collectively, the additional multimedia content in the a-photobook could be used as prompts for social interactions by supporting more immersive stories of the experiences to others. As participant R4 commented:

[a-photobook] just opens up your options a bit more. Like obviously I've got all the memories and everything up here so the way I look at it, it's like oh yeah I've got all of that but other people don't have that and that is where that augmentation comes in because you can show them that stuff.

### Enriched memories

Participants felt that the augmentation added a greater poignancy and control over the memories represented in their a-photobook through the additional multimedia they could chose to share, or not, with others. Without the Player app, the a-photobook appears like a traditional printed photobook and the link types cannot be accessed. Consequently, link types could be curated entirely for the private use of the owner/creator without fear that these could be assessed by accident by another viewer. This highlighted a novel a-photobook dichotomy where the a-photobook encompasses both ‘private’ and ‘public’ aspects. For example, participant R3 explained how she curated her a-photobook for personal enjoyment: ‘I decided to write it all in Dutch and not in English, because this is for me and not for you’. Furthermore, although most participants employed audio clips to added complementary sound to printed photobook images, participant R3 linked an unrelated audio clip to a random page in his a-photobook as it was part of an in-joke with his partner.

Moreover, as the additional digital media link types initially appear invisible without the Player app, participants could forget what they had embedded over time, leading to delightful surprises when revisited. Participant R6 addressed this when discussing her enjoyment of the a-photobook:

It’s that discovery. Yes, I’ve created that experience already, so I know that there’s going to be stuff that I want to enjoy. So, with the experience of looking at it and consuming that media, I would like that to remain a bit of surprise. You leave little Easter eggs for yourself to remember and rediscover.

The additional multimedia could also amplify the sentimental value of the original printed photobook and contextual richness of its displayed images by expanding the ways in which they could be represented and interacted with through different types of media, as supported by participant R7’s remark:

It brings the memories to life much more than just a simple photo album, so it brings the memories back to life in a kind of immersive, it’s really captivating, you’re experiencing it again, you’re experiencing the sounds and the voices, the feelings. It brings the feelings back more readily than just looking at pictures.

As well as representing visual aspects of previous experiences, through its augmentation, the a-photobook allowed participants to link images to additional online information and other digital media that showed how depicted scenes moved and sounded from the creator’s point of view. As put by participant R6, ‘[the a-photobook] adds texture to the experience in a way that you can’t at the moment’.

The a-photobook also appears to revitalise and improve accessibility to collected digital content on smartphones or other devices by providing a physical prompter to easily resurrect and relieve memories through associated multimedia: ‘I think having something that’s designed for the purpose of being this fusion kind of memory of your trip or your experiences. It’s so much nicer that having it on your phone…’ (R10). Notably, eight of nine participants spoke about the importance of having something physical to touch and display to prompt memory recollections of sentimental events while seven of nine discussed the less immediately accessible nature of digital media. The a-photobook therefore appeared to provide a physical bridge for reminiscing with curated digital media which encouraged participants to absorb themselves in the creative process of making it special:

it was just the fact that I know they’re [photos] not going to be sat, I knew that they weren’t going to be sat on my phone forever and never really be looked at, it made me put more effort in to the pictures to try and make them, try and get good ones (R3)

### Enhanced social interaction around photos

The completed a-photobooks were reported by all nine participants to encourage more social engagement from others during their reading and creation as the additional multimedia provided several approaches for sharing moments with others that could be viewed with or without the photobook. This highlighted another dichotomy introduced by the a-photobook: ‘passive’, and ‘active engagement’. ‘Passive engagement’ in this context refers to how the a-photobook behaves as a regular photobook and can thus support the social exploration of a physically photobook with others. As discussed by participant R2:

To me there’s something much more real about having the book, it takes me back to when you would sit side by side with someone and you’d flip through the pages and you’d pause, and it’s just not the same experience swiping on a phone, it’s much – for me it’s much more communal, maybe convivial to sit next to somebody with a photo album and turn pages together.

When the audience interacts with the complete a-photobook, this becomes ‘active engagement’ as its initially hidden digital elements are discovered and can be continuously modified by creating different a-book files linked to the same photobook using the NGP Authoring app. Consequently, the a-photobook could facilitate multiple family members and/or friends contributing to the same a-photobook, encouraging a more socially interactive reading experience, as acknowledged by participant R3’s comment:

I think if you would have this book and do the digital version, you do it as a family. You could also choose for each family member to do their own version of the same book [laughs]. So, it would be still personalised but also a family thing.

Moreover, friends and/or family members could actively work together to create and curate collective multimedia content for their joint a-photobook. This was a natural occurrence for six out of nine participants were either a partner or family member took an active interest in the creation of the a-photobook and began collecting and curating content alongside the participant while they were on holiday. For example, Participant R1 affirmed the contribution of her partner by mentioning, ‘it was definitely a group effort because we talked about which things, we really wanted to have in it’ while participant R4 discussed the specific roles he and his partner undertook while creating and curation content: ‘she was more keen about it, especially the videos part, she wanted to capture more of the videos, and I wanted to capture more on the images side of things’ (R4).

Lastly, four out of nine participants suggested that the a-photobook approach could offer a way of socialising smartphones through their employment to access content from physical printed material in the real world to share with others. For example, participant R1 overtly appreciated how relevant multimedia could be archived through the Player app, making it readily accessible to view with others without the physical book:

You can use it without actually having the physical photo bit. It’s been nice being in situations where I’ve not had the photo bit with me, and I’ve wanted to show people stuff… On the train the other day, [partner] and I were just going through some of it, as like a nice kind of reminder of the trip, and yeah to show friends and family what we got up to.

Participant R2 also reflected on how the a-photobook changes the social perception of smartphones, which are generally perceived as private and for personal use:

If you would be sitting with your phone I wouldn’t [say] what are you looking at? Whereas if I would be like this [with an a-photobook], I think it’s more inviting to and also again more socially acceptable to have a look as well.

## Discussion

Printed photography continues to play a significant social role in daily life, through its assembly into photobooks. However, this a highly subjective and socially influenced practice [36] where images are curated to represent ideals of a happy personal or family life, [37] and natural changes in memory can transform their original associations overtime [38]. In this study, we have found that augmented photobooks add further complexity and realism to the traditional photobook. They do this through embedded digital media, automatically hidden from view, creating multi-layered communication opportunities for conceptualising self. We have found that this enables more spontaneous and realistic and/or personal renditions of everyday life that are not necessarily curated for public viewing, generating more individually meaningful narratives. Participants also recognised that the same a-photobook could also hold a multitude of views and perspectives (annotations) from several individuals simultaneously, to represent collective experiences in a new way. These annotations could be added at the original time of authoring through the Authoring App, or later when viewing content through the Player app enhanced to support ‘reader annotations’. This realisation was also made apparent from participants’ desire for future iterations of the Authoring App to have a group feature that would allow multiple members of a family or friendship group to create and add to the same augmented photobook simultaneously.

A-photobooks therefore seem to offer alternative means of self-expression to the posting of photos online. Online personal images are carefully curated according to assumed social conventions [39] and their distribution can lead to neurotic checking behaviour for cues of social acceptance [40]. In contrast, a-photobooks offer a different offline avenue for self-presentation that appears to escape this level of ‘social surveillance’ [41] through the privacy of its closed system. For example, neither the printed images or linked media are published online and are only available through the Player app on the user’s personal device and/or interactions between it and the physical photobook—although external websites can be linked to the a-photobook. Security and privacy here is assured through possession of the physical printed photobook, and even copies of the same photobook could be made unique through their linking to alternative digital media. Consequently, novel sharing possibilities and controls are introduced by the a-photobook technology and content which could be configured or designed to be more or less private, depending on the author’s wishes.

These interactive possibilities go far beyond the mere printing of passive digital stories comprising images, sounds and video. Our participants discovered for themselves that browsing an a-photobook is not like playing a linear multimedia slide show, but rather affords multiple journeys through different kinds of optional hotlinks associated with printed pages. They liked the fact that they could chose to reveal or hide these links when showing their creations to other people face-to-face, but that opened up questions about who holds the a-photobook and who holds the phone on these sharing occasions. Indeed, the realisation that the same book could be browsed simultaneously with multiple phones owned by each member of the family or sharing group was a surprise discovery of the study. Annotations currently propagate across phones when users download the same a-photobook file to their phone for viewing in the Player app, but authors could set constraints on this, or even vary annotations for different audiences. Further annotations added by readers through the Player app would currently stay on their phone only, but could be designed to propagate to other phones. Notably, these were speculative reflections from participants on future possible interactions with the a-photobook rather than activities they undertook during the study. Nonetheless, this demonstrates potential for a-photobooks to support an interplay of printed and digital content across multiple physical and electronic devices, leading to a fascinating ‘phygital’ nexus of possibilities for new forms of media sharing, reminiscing and storytelling [42].

In short, the a-photobook supports a new way reliving and telling stories of past moments, specifically as a physical photobook and as an archiving tool for associated digital content. Its physicality, including its digital aspects, makes it more visible, accessible and attractive for immediate engagement. Given that the handling of physical images can elicit emotional connections to themes depicted [10] and imaginings of related experiences [43], associated digital media can be played to add further contextual richness and sensory stimuli to these responses. Ambient sound with printed image was found again by participants to enhance memory, as in previous work (e.g. [44]) while video added realism and detail (e.g. [9]). Links to factual information such as the websites of accommodation, restaurants, or visitor attractions also supported memory, but went beyond this to facilitate re-visiting places by travellers or by others to which they would recommend them. Hence, memories were enriched and extended by digital links but also brought into the open by the printed photobooks as tangible reminders of journeys made and roads less travelled, through their physical presence in the home.

While a-photobooks are unlikely to revive the traditional family album as an idealistic portrayal of family life, they do show potential for being easily integrated into existing photo-sharing practices for special events around the emerging photobook format. Both the Authoring App and Player app’s reported ease-of-use allow that format to be extended with additional multimedia content from smartphones or social media platforms, through novel blends of digital and print affordances and interactions. These are unlocked by authors simply annotating printed pages with digital materials, and by readers simply turning pages and selecting associated links to play, on both the ubiquitous smartphone alongside the book. Although minor adjustments were suggested to improve the overall user experience of this interaction, creating a-photobooks in this way appears to enable the smartphone itself to become the vehicle for augmenting the photobook beyond its current limitations and constraints, for greater enjoyment, conversation, and control.

## Conclusions

The a-photobook presents a promising alternative to the tradition photo album where users can augment pre-existing or purpose-made photobooks with complementary multimedia to further expand its associated meanings and presented narratives. It connects currently separate physical and digital photo sharing practices, such as generation of bespoke photobooks using online platforms and personal multimedia sharing on smartphone devices. This appears to deliver a novel way of presenting self that allows for multiple layers of access and privacy of personal content not currently possible using these elements in isolation. Further research and development is required to allow for more incremental addition of digital content over time, and explore requirements for gifting and sharing a-photobooks within family or friendship groups with different levels of reading and writing access. We recommend this to be an interesting departure from current social media and digital storytelling systems, which brings back the beauty and tangibility of printed photographs as a more controllable interface to personal digital media.

## Conflict of interest

The authors declare no competing interests.

## Data Availability

The datasets generated during and/or analysed during the current study are not publicly available due to the sensitive nature of the content (i.e. multimedia depicting personal family holidays), but additional select anonymised examples are available from the corresponding author on reasonable request.
